# Multiplexed Immunosensor Based on the Amperometric Transduction for Monitoring of Marine Pollutants in Sea Water

**DOI:** 10.3390/s20195532

**Published:** 2020-09-27

**Authors:** J.-Pablo Salvador, Klaudia Kopper, Andrea Miti, Ana Sanchis, M.-Pilar Marco

**Affiliations:** 1 CIBER de Bioingeniería, Biomaterialesy Nanomedicina (CIBER-BBN), Jordi Girona 18–26, 08034 Barcelona, Spain; kkonqb@cid.csic.es (K.K.); andrea.miti@unibo.it (A.M.); ana.sanchis@iqac.csic.es (A.S.); pilar.marco@cid.csic.es (M.-P.M.); 2 Nanobiotechnology for Diagnostics group (Nb4D), IQAC- CSIC, Jordi Girona 18–26, 08034 Barcelona, Spain

**Keywords:** multiplexation, amperometric biosensor, seawater, environmental monitoring

## Abstract

Environmental pollutants vigilance is one of the main problems that the aquaculture industry has to face with the objective to ensure the quality of their products and prevent entrance in the food chain that finally may arrive to the consumer. Contaminants such as hormones, antibiotics or biocides are especially relevant due to their toxicity, pharmacological effect or hormonal activity that can be considered harmful for the final consumer. The contaminants can be detected in the environment where the food is growing, and their concentration can be found (i.e., seawater) in the range of µg·L^−1^, ng·L^−1^ or even in lower concentrations. Thus, sensitive and selective methods for their monitoring are required to avoid their arrival in the food chain. Here, the development of a multiplexed amperometric biosensor is described, based on the use of specific antibodies to reach the necessary detectability to measure the targeted contaminants directly in seawater. The multiplexed immunosensor allows the detection of four relevant pollutants, such as el Irgarol 1051, sulfapyridine, chloramphenicol and estradiol, reaching an IC_50_ of 5.04 ± 0.29, 3.45 ± 0.29, 4.17 ± 0.44 and 5.94 ± 0.28 µg·L^−1^, directly measured in seawater.

## 1. Introduction

Aquaculture activities are becoming one of the pillars in the sector of worldwide food production, being classified as the fastest growing food source of aquatic animals. In order to ensure the sustainability of that industry, it is mandatory to establish reliable monitoring programs and analytical tools which may allow the real-time control/monitoring of physical chemical parameters as well as certain inorganic and organic contaminants. Current legislation such as the Water Framework Directive (WFD, 2000/60/EC) in parallel with the Marine Strategy Directive (2008/56/EC) have raised concern about environmentally emerging pollutants, such as pharmaceuticals and endocrine-disrupting chemicals (EDC) [1,2,3]. Even though there are several commercially available devices that can monitor variables such as pH, pO2, temperature, salinity, nitrites and ammonia, among others, there is a lack of devices capable of quantitative detection of organic residues in the environmental field. Within organic pollutants, some classes of contaminants have a high presence in water environments, such as household chemicals, industrial by-products, like phthalates [4], pesticides [5] or personal care products [6], together with the mentioned pharmaceuticals or EDCs. 

Nowadays, the determination of these types of compounds has been reported employing chromatographic techniques, usually coupled to mass spectrometry (MS) detectors, which are able to reach really low detection limits [7,8,9,10]. The presence of organic pollutants in different kinds of water such as river, tap and seawater, or even in effluents of waters from sewage treatment plants, are reported to be in the order of µg·L^−1^, ng·L^−1^ or even in lower concentrations. The required limits of detection can often be achieved thanks to the incorporation of a preconcentration step prior to the analysis, which allows the collection of different pollutants in a large amount of sample, followed by their elution in a small portion of solvent [11]. The advantages of the chromatographic techniques used for environmental vigilance are widely described in the literature, and are based on their high detectability, multi-analyte determination capacities and their good accuracy and reproducibility levels [12,13]. On the other hand, those technologies are usually considered expensive and time-consuming, with high requirements in terms of equipment and highly qualified personnel. The need for preconcentration/clean-up steps to improve the detectability and the lack of harmonized protocols for the enrichment of the samples are some of the drawbacks presented by these techniques. Nowadays, portable MS spectrometers have been developed, mostly based on ambient ionization which only allows the analysis of volatile and semi-volatile organic compounds [14,15] from air and aqueous sample matrices. Moreover, the analysis of low- or ultra-low-concentrated pharmaceuticals or other contaminants is still a challenge for on-site monitoring using these techniques. 

Antibody-based assays are excellent alternatives to be used as screening tools to face the high number of analyses due to their advantages, such as high levels of sensitivity, wide ranges of selectivity and simple and high-throughput protocols which can be applied in the environmental field [16]. All those characteristics make them ideal candidates to be implemented as screening tools. In the last three decades, several antibodies have been raised for the development of immunoassays for the detection of environmental pollutants either for Enzyme Linked Immunosorbent Assay (ELISA), lateral flow immunoassays or immunosensors [17,18,19]. On the other hand, the development of antibody-based techniques may be expensive and may require a long development. Other drawbacks such as instability, non-specific interaction with matrices and certain animal variability can be minimized and solved [20]. 

Concerning the multiplexation capabilities, they are sometimes limited by the availability of immunoreagents for the compounds to be determined or phenomena like shared and cross-reactivity inherent from some antibodies [21]. During the last few years, different multiplexed systems for the environment contaminants have been reported [22]. Nevertheless, among these systems, multiplexed analytical biosensors based on electrochemical detection for environmental contaminants have not been successful. In general, multiplexing for the detection of multiple analytes using electrochemical signals is still a challenge. Electrochemical immunosensors using impedance transducers are based on capacitance/resistance changes occurring at conductive or semiconductive surface, and amperometric transducers which are based on the use of electroactive mediators as the substrate of antibody-labeled enzymes, such as horseradish peroxidase (HRP), alkaline phosphatase (AP) or glucose oxidase [23]. As an example, impedimetric transducers solve the multiplexing drawback using a site-encoded location for the different analytes to be tested. However, impedance-based immunosensors need to be deeply studied to be more robust and to avoid nonspecific interferences from real samples [24]. On the other hand, amperometric immunosensors used the same strategy but are limited by the crosstalk that can exist in the different sensors if they are place in the same sensor chip [25]. A few examples can be found which employed multiplexed devices using amperometric signals [26,27], however most of them are based on the use of magnetic particles which require different immunochemical steps outside of the sensor electrode. In this work, we aim to present solutions for the determination of relevant environmental pollutants through the development of a multianalyte amperometric immunosensor, immobilizing the corresponding immunoreagents in the surface of a gold screen-printed electrode. The proposed immunosensor allowed the selective multiplexed determination of up to four relevant environmental analytes directly in seawater in flow mode.

## 2. Experimental Section

### 2.1. Materials and Instrumentation

Amperometric measurements were performed with a multipotentiostat µSTAT 200 potentiostat (DropSens, Spain). Au screen-printed electrodes (SPE 8× DRP-8X220AT, DropSens, Spain) consisting of a 2.5 mm smooth Au working electrode, an Au counter electrode and an Ag pseudo-reference electrode, were used. A batch cell made on polymethylmethacrylate support (PMMA support) with 8 wells, designed and manufactured by Micro-Nano Technologies Unit, of the Unique Scientific and Technical Infrastructures (U8 of the ICTS “NANBIOSIS”) from the Institute of Microelectronics of Barcelona (IMB-CNM, Barcelona, Spain), was used. A flow-cell (FLWCL8X, DropSens, Spain) for screen-printed electrodes was used for flow experiments. A UV/Ozone Procleaner™ unit from Bioforce Nanoscience (Ames, IA, USA) was used to clean the surface of the electrodes. The peristaltic pump was purchased from ISMATEC (Model ISM404B, Wertheim, Germany) and the corresponding tubing was made in Tygon with an inner diameter of 0.75 mm. The tubing that connects the peristaltic pump and the flow cell is made of Polytetrafluoroethylene (PTFE) with an inner diameter of 0.5 mm. The pH and the conductivity of all of the buffers and solutions were measured with a pH meter, pH 540 GLP, and a conductimeter LF 340, respectively (WTW, Weilheim, Germany). The calibration curves were fitted to a four-parameter logistic equation using the GraphPad Prism 5 software (GraphPad Software, San Diego, CA, USA). IC_50_ corresponds to half maximal inhibitory concentration. The limit of detection (LOD) and the limit of quantification (LOQ) are defined as the concentration producing 90% (IC_90_) and 80% (IC_80_) of the maximal signal, respectively. 

### 2.2. Chemicals and Biochemicals

The immunoreagents used in this study for the detection of Irgarol 1051 [28,29] and sulfonamides [30], 4e-BSA/As87 and SA2-BSA/As155 respectively, have been previously described. The immunoreagents used for the detection of Chloramphenicol, CA6-BSA/As226, were prepared in-house. Additionally, the antibody R69 for the detection of estradiol was kindly provided by Prof. R. Quidant (ICFO, Spain) and the corresponding coating antigen 6E2_4-BSA was prepared by the active ester method, as described in Reference [31]. Specifically, all antibodies used were produced in rabbits and all coating antigens used are Bovine Serum Albumin (BSA) conjugates as a carrier protein. All the coating antigens have been produced with the support of the ICTS “NANBIOSIS”, more specifically by the Custom Antibody Service (CAbS, Centro de Investigación Biomédica en Red de Bioingeniería, Biomateriales y Nanomedicina (CIBER-BBN), Institute of Advance Chemistry of Catalonia from the Spanish Council for Scientific Research (IQAC-CSIC)). The secondary goat anti-rabbit IgG peroxidase conjugate (Anti-IgG–HRP) was purchased from Sigma (St. Louis, MI, USA). The O-(2-Carboxyethyl)-O’-(2-mercaptoethyl)-heptaethylene glycol (PEG-thiol-acid) and 2,5,8,11,14,17,20-Heptaoxadocosane-22-thiol (mPEG-thiol) were acquired from Polypure (Oslo, Norway). Stock solutions of Irgarol 1051, sulfapyridine, chloramphenicol and estradiol (10 mmol·L^−1^) were prepared in dimethyl sulfoxide (DMSO) (Merck) and stored at 4 °C. Artificial seawater (aSW) was purchased from Sigma Chemical Co. (St. Louis, MO, USA) and prepared at 40 mg·mL^−1^ in Ultrapure water.

### 2.3. Buffers and Solutions

Phosphate-buffered saline (PBS) is 0.01 M phosphate buffer on a 0.8% saline solution, with a pH of 7.5. PBST is PBS with the addition of 0.05% Tween 20. PBT-2× is 0.02 M phosphate buffer at pH 7.5 and 0.1% Tween 20. Artificial seawater was prepared by dissolving sea salts (Sigma, St. Louis, MI, USA) according to the supplier specifications at 40 g·L^−1^. For electrochemical measurements, citrate buffer was used at 0.04 M, pH 5.5. The substrate solution consisted of 0.001% TMB (3,3′,5,5′-tetramethylbenzidine) and 0.0004% H_2_O_2_ in citrate buffer 0.1 M potassium chloride (KCl). The regeneration solution was 0.3 M sodium hydroxide (NaOH).

### 2.4. Biofunctionalization Protocol

The protocol of immobilization was previously described [32] and adapted for the actual SPE 8× format. The gold SPEs were rinsed gently with water and ethanol and then dried. Afterwards, the SPEs were cleaned using an UV/Ozone Procleaner™ for 15 min. The activation of the gold surface was accomplished through a mixed self-assembled monolayer (m-SAM) prepared by pumping an ethanolic solution of 1 mL of 2.5 mM of PEG-thiol-acid and 7.5 mM of mPEG-thiol at 40 μL·min^−1^ through the active gold surface area of SPEs. The chips were then gently washed with ethanol and dried. Subsequently, the biofunctionalization of the SPE with the corresponding hapten bioconjugates was performed by the addition and mixing of a 25 µL mixture of 1-Ethyl-3-(3-dimethylaminopropyl)carbodiimide/N-Hydroxysuccinimide (EDC/NHS, 200 mM in PBS) and 25 µL of the appropriate concentration of the coating antigen in PBS, which was incubated for 3 h at room temperature (RT). The different hapten bioconjugates were immobilized per duplicate per chip. After the time of the incubation, the SPEs were rinsed with PBS and the remaining activated carboxylic acids were capped by adding a solution of ethanolamine (1 M in PBS). Finally, the biofunctionalized SPE chips were washed with water and stored in a desiccator until use.

### 2.5. Immunosensor Protocol (Static Mode)

The functionalized immunosensor gold SPE 8× was placed in a batch cell. Different SPE from the SPE 8× chip were coated with the different coating antigens at 100 µg·mL^−1^. After the functionalization, the corresponding solutions of the antibodies (1/1000 diluted in PBST, 100 µL·well^−1^) were added. After 30 min at RT, the SPE 8× was washed with PBST (3 × 300 µL) and the anti-IgG-HRP solution (1.24 µg·mL^−1^ in PBST, 100 µL·well^−1^) was added and incubated for 30 min more at RT. The SPE 8× electrode chip was washed again and the substrate solution was added (100 µL·well^−1^). The chronoamperograms were acquired and the final current was obtained after stabilization of the signal. The SPE 8× electrode chip was regenerated by the addition of 0.3 M NaOH for 30 min (100 µL·well^−1^). After that, the SPE 8× electrode chip was washed again and ready for the next assay.

### 2.6. Immunosensor Protocol (Flow Mode)

The selected protocol used a flow mode with a flow rate of 100 μL·min^−1^ during the whole procedure, as previously described [32] with slight modifications. All the steps have been performed at RT. First, the SPE chip was placed into a flow cell. Prior to the analysis, the standards/buffer/sea samples were prepared in 4 mL and were mixed with 4 mL of the antibody solution (single or cocktail of antibodies) at the appropriate concentrations in buffer PBST 2×. The mixture was split in eight different channels and was flowed into each individual sensor cell for 10 min followed by PBST buffer (5 min). Then, 8 mL of the anti-IgG-HRP solution (1.24 µg·mL^−1^ in PBST) was flowed into the different channels for an additional 10 min, followed again by PBST buffer (5 min) for washing. The chip was then conditioned in citrate buffer during 5 min, and afterwards, the first chronoamperogram was recorded. The substrate solution was then flowed for 3 min, and then, the second chronoamperogram was recorded. The chip was then washed with citrate buffer (5 min) and finally, regenerated by flowing 0.3 M NaOH (10 min) followed by PBST buffer (5 min). The chip was then ready for the subsequent analysis.

### 2.7. Amperometric Measurement

The chronoamperograms were acquired at an applied potential of −0.10 V versus the Ag pseudo-reference electrode [33] during 60 s for both batch and flow mode. The signals acquired in a flow mode were recorded at the flow rate described above. The recorded signal was the mean value of the current obtained in the last 20 s when the steady-state was reached. The difference between the amperogram obtained when the substrate solution (S) is flowed and the amperogram recorded with the citrate buffer only (C), is considered the specific signal produced by the binding of the anti-IgG-HRP bioconjugate.

## 3. Results and Discussion

### 3.1. Biofunctionalization of SPE

The biofunctionalization protocol used in this work is based on a previous work performed by our group [32]. The protocol takes advantage of the use of a mixed self-assembled monolayer (SAM) consisting of a mixture of heterobifunctional PEG molecules, that have a thiol group on one side, and a carboxylic acid/methoxy functional group on the other side. The use of PEG-based compounds for the derivatization of surfaces has been widely used to improve the hydrophilic nature and better coverage of the surface and to provide a better spatial distribution of the carboxylic acids for optimal coupling of the proteins [34]. 

**Figure 1:**
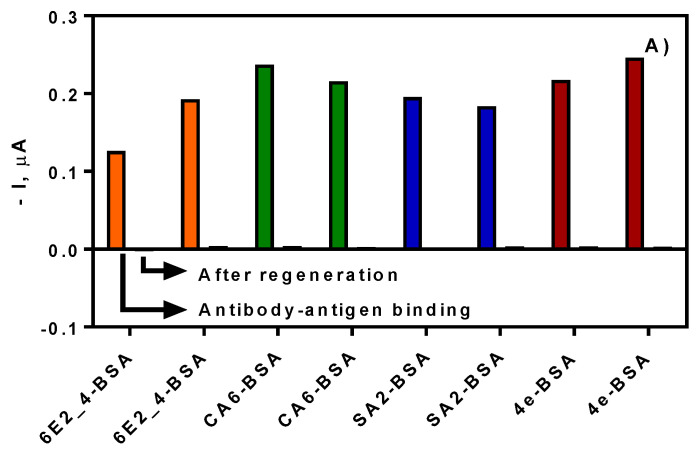

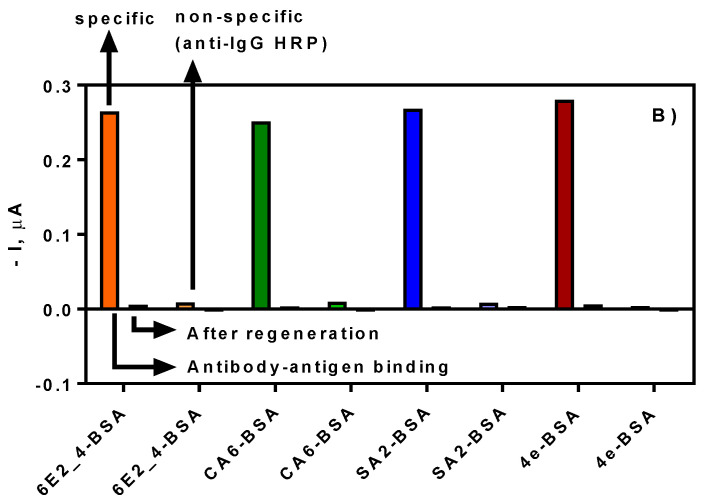
(**A**) Current signal obtained from each channel in a batch cell. Each coating antigen was immobilized at 100 µg·mL^−1^, and the corresponding specific antibody was added (100 µL·SPE^−1^ at 1/1000 in PBST). Anti-IgG HRP conjugate was added to every channel of the SPE (100 µL·SPE^−1^ at 1.24 µg·mL^−1^ in PBST). Regeneration was carried out adding NaOH 0.3 M solution (100 µL·SPE^−1^). (**B**) The same functionalized chip was tested again in the same conditions but adding only PBST (100 µL·SPE^−1^) on one SPE of each pair, in order to test the non-specific binding of anti-IgG HRP conjugate (100 µL·SPE^−1^ at 1.24 µg·mL^−1^ in PBST).

The immobilization of the corresponding coating antigens was performed through the carbodiimide chemistry using a mixture of EDC/NHS. The use of those reagents allows the chemical activation of the carboxylic acids from the heterobifunctional PEG molecules, forming an active ester which will react with the free amino groups of the different biomolecules to immobilize, i.e., BSA conjugates [32,35,36]. The SPE 8× was used for the development of the multiplexed immunosensor, in which each coating antigen was immobilized per duplicate. Initially, a concentration of 100 µg·mL^−1^ was used for each coating antigen on the different working electrodes in order to demonstrate the correct implementation of the biofunctionalization protocol. The assay took place in a batch cell following the static mode protocol for the initial evaluation of the antigen-antibody binding. In all cases, corresponding current was obtained, demonstrating the successful modification of the different electrodes (see Figure 1A). After that, a regeneration step was carried out adding a solution of NaOH 0.3 M and the current was measured again adding the substrate solution. As it can be observed from Figure 1A, the regeneration solution broke the interaction between the antigen–antibody interaction, allowing the addition of the next antibody solution on the same chip. In Figure 1B, a second round of the corresponding antibody was added, but testing in one of them only the contribution of the anti-IgG HRP conjugate as a nonspecific signal. Comparing the specific and the nonspecific signal, it can be observed that no significant signal was provided by the secondary antibody HRP conjugate alone. Again, any signal was observed after the addition of the regeneration solution. The gold screen-printed electrodes allowed, on one side, the covalent immobilization of the coating antigen, and on the other side, the easy regeneration of the SPE for the next measurement.

### 3.2. Individual Immunosensors for the Detection of Irgarol 1051, Sulfapyridine, Chloramphenicol and Estradiol

**Figure 2:**
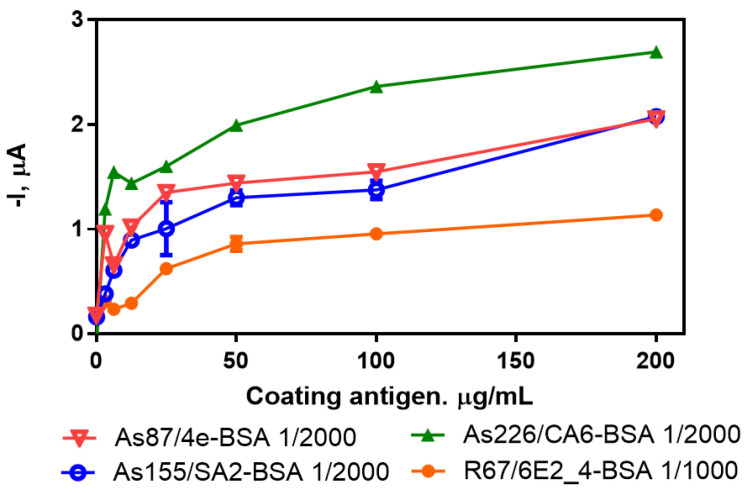
Titration experiment for the coating antigens 4e-BSA, SA2-BSA, CA6-BSA and 6E2_4-BSA and the corresponding antibodies As87 (1/2000 in PBST), As155 (1/2000 in PBST), As226 (1/2000 in PBST) and R67 (1/1000 in PBST), respectively.

One of the important issues in any immunochemical technique for the detection of small molecules like this multiplexed sensor is to find the optimal concentrations of immunoreagents involved in the detection of each specific target pollutant. This selection was based on a two-dimensional (2D) checkerboard titration experiment [37], in which different concentrations of the coating antigen and different concentrations of the antibodies were tested. In order to accomplish that, four different gold SPE 8× electrodes were prepared with their corresponding antigens with a range of concentrations of 200, 100, 50, 25, 12.5, 6.25, 3.125 and 0 µg·mL^−1^. Different concentrations of each corresponding antibody (1/1000, 1/2000 and 1/4000 dilution factor in PBST) were added to the gold SPEs and the amperometric signal was recorded using the flow mode protocol After that, a set of saturation curves were obtained for each pair of immunoreagents (see Supplementary Figure S1). The criterion for the selection of the most appropriate concentration of the coating antigen was to choose the concentration that reaches the 70% of signal of the saturation curve [38]. The antibody concentration was also selected taking into consideration a signal of approximately –1.0 µA, which corresponds to antibody dilutions of 1/2000 for As87, As155 and As228, and 1/1000 for R64 (see Figure 2). On the basis of these criteria, the optimal concentrations of coating antigens to be immobilized on the surface of the SPE were 10, 25, 10 and 50 µg/mL for 4e-BSA, SA2-BSA, CA6-BSA and 6E2_4-BSA, respectively (see Table 1).

**Figure 3:**
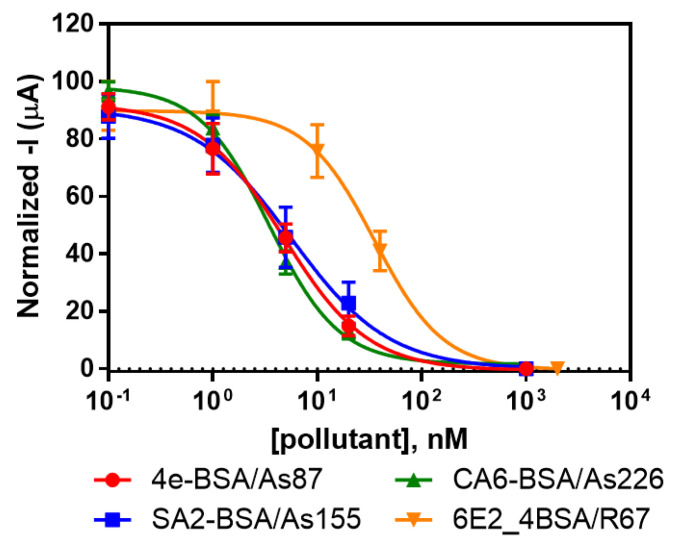
Calibration curves for the individual immunosensors on gold SPE 8× electrodes. The figure summarizes the normalized data for each assay obtained on different days (N = 9, 6, 9 and 3 for Irgarol 1051, sulfapyridine, chloramphenicol and estradiol assays, respectively).

The same protocol of analysis was used in the past for the detection of Irgarol 1051 [32] and Deltamethrin [36] using individual gold SPEs. In this work, the analysis was performed for each pair of immunoreagents using the SPE 8× sensor. The selected concentrations for each coating antigen were immobilized on the gold SPE using the same strategy explained before. After that, each immunosensor was tested individually in order to evaluate the achievable detectability. Initially, different concentrations of the corresponding pollutants were mixed with the antibody, and, after flowing the mixture through the immunosensor, the signal was acquired. The results revealed a good concordance between the signal acquired and the spiked concentration for each pollutant (see Figure 3). The IC_50_ values reached were 1.20 ± 0.23, 1.40 ± 0.39, 1.12 ± 0.37 and 9.72 ± 0.37 µg·L^−1^ for Irgarol 1051, sulfapyridine, chloramphenicol and estradiol, respectively. The results obtained with the immunosensor were similar to the results obtained by ELISA and fluorescent microarray in a previous characterization of the selected immunoreagents [11,31]. Moreover, our group has developed other immunosensors exploring different transducing principles with similar detectability for sulfonamides and Irgarol [39,40,41].

**Figure 4:**
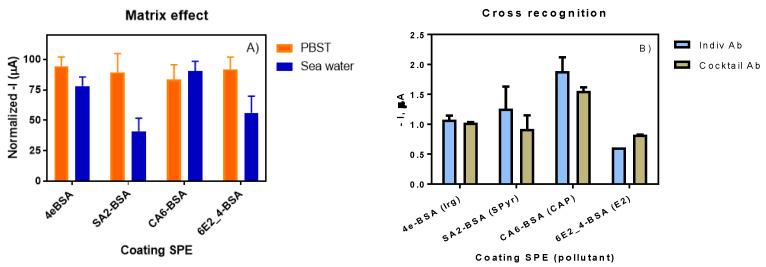
(**A**) Matrix effect of seawater compared with buffer for each individual assay (N = 4). (**B**) Comparison between the signal obtained from the individual assay (coating antigen versus its corresponding antibody) and the multianalyte assay (coating antigen versus a cocktail of antibodies).

The matrix selected for the analysis of the targeted pollutants was seawater. Seawater is characterized by its high salinity content of 3.1–3.8% (approximately 50 mS·cm^−1^) and a pH between 7.5 and 8.4. Those parameters should be considered in order to study the matrix effect concerning the antigen–antibody recognition. In our previous work [31], the matrix effect for all the immunoreagents was tested demonstrating to be minimum in all cases, allowing the direct measure of seawater. Therefore, it was decided to test the matrix effect of seawater in the immunosensor format. As it can be observed in Figure 4A, the assays 4e-BSA/As87 and CA6-BSA/As226 were not affected by the seawater, but in the case of SA2-BSA/As155 and 6E2_4-BSA/R67, a partial inhibition of the signal was observed. However, since a meaningful response was detectable, we can conclude that the effect of the seawater did not impede the correct development of the antigen–antibody interaction, and therefore, the immunoassay could be carried out without any significant problem. 

### 3.3. Multiplexed Immunosensor for the Detection of Irgarol 1051, Sulfapyridine, Chloramphenicol and Estradiol

The development of a multiplexed immunosensor required the use of a cocktail of antibodies for the detection of the different selected pollutants in parallel. An initial evaluation was required in order to demonstrate the absence of cross-recognition or shared reactivities of the different antibodies versus the coating antigens. This fact was previously assessed in our last work [31], where no substantial effect was observed using the different antibodies versus the unspecific antigens; however, in order to confirm the specific behavior previously shown, it was necessary to test this phenomenon in the immunosensor format as well. As it can be observed in Figure 4B, the difference between the use of the cocktail of antibodies versus the use of each specific antibody individually was minimal and can be considered negligible. 

**Figure 5:**
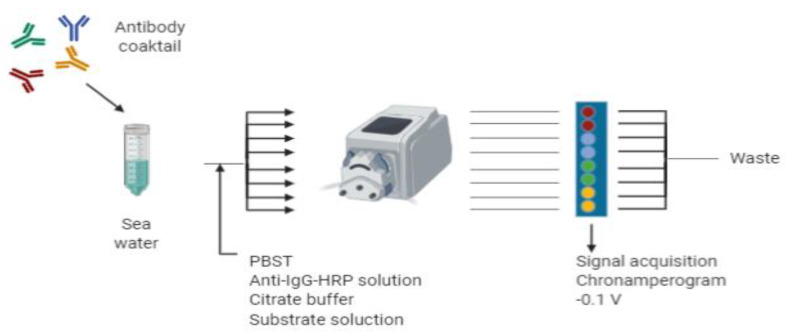
Schematic representation of the final multiplexed immunosensor platform for the determination the targeted pollutants in sea water.

The multiplexation of the amperometric measurement was designed using a site-encoded configuration in which each SPE was physically separated from the others using different microfluidic chambers (see Figure 5). This physical separation was necessary in order to avoid any possible crosstalk caused by the diffusion of the electroactive molecules. Some authors avoided the crosstalk between different working electrodes by performing the assay in the same batch cell, however a sequential acquisition of the signal for each electrode [26,42] is required or, alternatively, a different label for each antibody is needed [43]. Mixtures of each seawater sample and the cocktail of antibodies in the appropriate buffer were split in equal volumes and flowed through different microfluidic chambers. According to the multiplexed configuration of the SPE 8× (with two replicates for each coating antigen), a total volume of 8 mL was prepared (4 mL seawater sample + 4 mL cocktail of antibodies in buffer).

**Figure 6:**
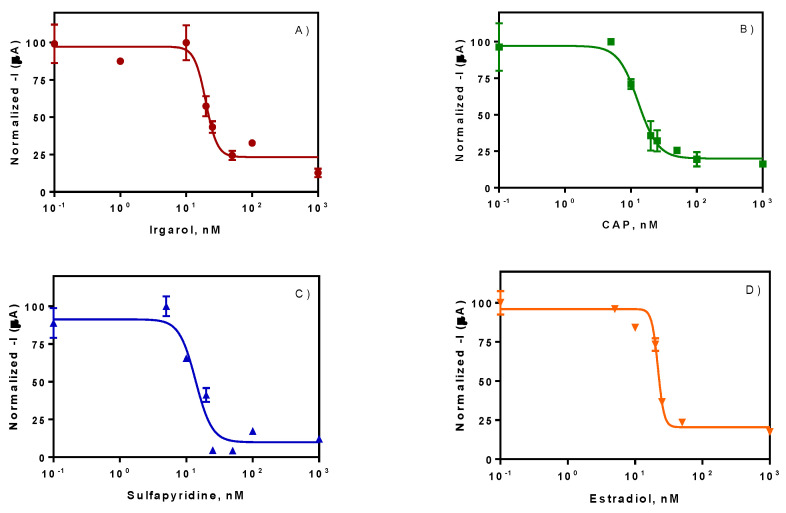
Calibration curves obtained with the multiplexed immunosensor on gold SPE 8× electrodes for Irgarol (**A**), chloramphenicol (**B**), sulfapyridine (**C**) and estradiol (**D**). The results summarize the normalized data for each assay obtained on two different days.

Spiked seawater samples at different concentrations were measured using the above-mentioned configuration, providing four calibration curves for the four analytes selected: Irgarol 1051, Sulfapyridine, chloramphenicol and estradiol (see Figure 6). As it can be observed, good detectabilites were achieved reaching IC_50_ values of 5.04 ± 0.29, 3.45 ± 0.29, 4.17 ± 0.44 and 5.94 ± 0.28 µg·L^−1^ for Irgarol 1051, sulfapyridine, chloramphenicol and estradiol respectively, directly measured in seawater. The results obtained in terms of IC_50_ and LOD are in good agreement with the results previously obtained in other platforms such as ELISA, fluorescent microarray or individual amperometric immunosensor (see Table 2). The total assay time was established in 38 min for the simultaneous signal acquisition from the eight functionalized SPEs. The developed platform is suitable for the simultaneous detection of four relevant contaminants per duplicate at the same time, directly in seawater and without any pretreatment of the sample.

## 4. Conclusions

This work gave a solution for the direct measurement of four relevant environmental pollutants in seawater. The site-encoded configuration of the eight gold screen-printed electrodes allowed the implementation of four competitive immunoassays with a cocktail of antibodies. Moreover, this configuration avoided the undesired crosstalk between all the screen-printed electrodes and the simultaneous measurement of the signal. The detectability achieved for each pollutant in the multiplexed immunosensor was in the range of µg·L^−1^ (IC_50_ of 5.04 ± 0.29, 3.45 ± 0.29, 4.17 ± 0.44 and 5.94 ± 0.28 µg·L^−1^ for Irgarol 1051, sulfapyridine, chloramphenicol and estradiol), directly measured in seawater with similar values compared to the individual immunosensor configuration, and also in good agreement with the corresponding ELISA assay. The total assay time was defined in 38 min allowing the simultaneous measurement of the eight functionalized SPEs. This smart system also allowed the regeneration of the chip, giving the chance to perform sequential analysis of different samples. The multiplexation was thus achieved in terms of detectability, selectivity and measurements in seawater matrix. 

## Figures and Tables

**Table 1:** Chemical structures and immunoreagents used for the determination of Irgarol 1051, sulfapyridine, chloramphenicol and estradiol. The concentration obtained from the two-dimensional (2D) checkerboard titration experiment is included.

Analyte	Irgarol 1051	Sulfapyridine	Chloramphenicol	Estradiol
**Structure**	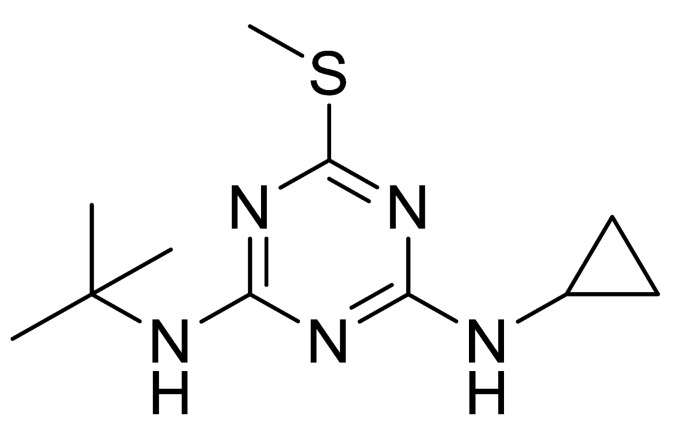	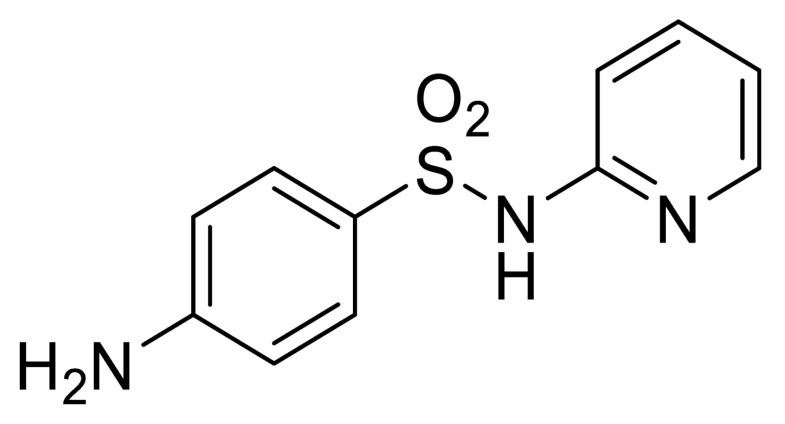	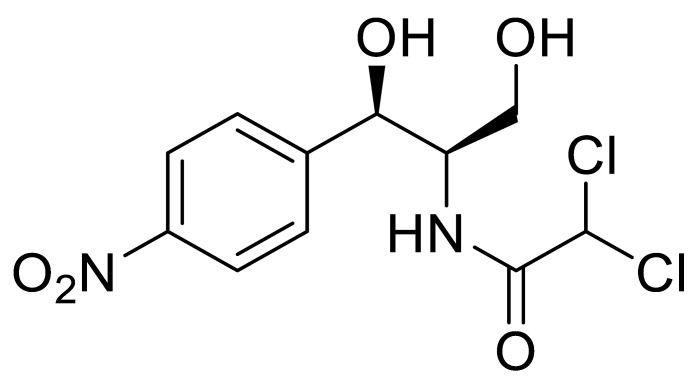	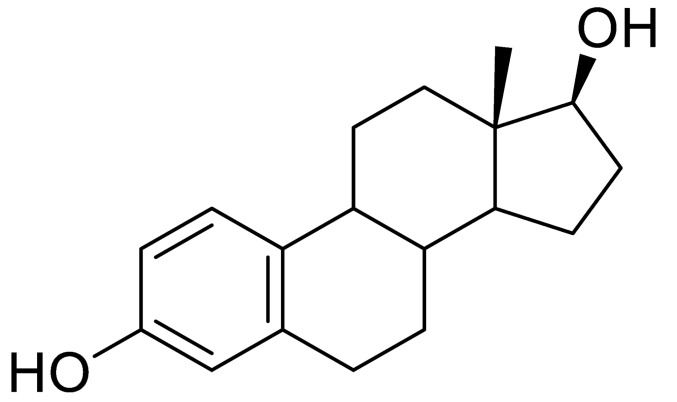
**Anti-serum**	As87	As155	As226	R64
**As dilution**	1/2000	1/2000	1/2000	1/1000
**Coating antigen (CA)**	4e-BSA	SA2-BSA	CA6-BSA	6E2_4-BSA
**(CA), µg/mL**	10	25	10	50
**Reference**	[28]	[30]	-	-

**Table 2:** Analytical parameters of the calibration curves obtained from individual and multianalyte immunoassays on gold SPE 8x compared with the parameters previously evaluated by ELISA [11], fluorescent microarray [31] and individual SPE in 1× format [32].

	ELISA [11] *^1^	Microarray [31] *^1^	SPE 1x Format [32] *^2^	SPE 8× Format *^1^	Multianalyte SPE 8× Format *^2^	ELISA [11]	Microarray [31]	Individual SPE 8× Format	Multianalyte SPE 8× Format
	**Irgarol 1051**					**Sulfapyridine**			
**Signalmax**	100.4 ± 0.4	100.6 ± 2.1	95.42 ± 4.40	92.2 ± 6.0	97.3 ± 6.5	100.4 ± 0.6	97.1 ± 5.1	90.9 ± 10.0	91.3 ± 4.3
**Signalmin**	2.9 ± 0.4	3.1 ± 1.9	−2.45 ± 4.78	−0.6 ± 5.2	23.3 ± 8.3	2.7 ± 1.2	−2.5 ± 6.4	−0.22 ± 8.4	9.9 ± 7.0
**Slope**	−0.98 ± 0.22	−1.45 ± 0.19	−0.81 ± 0.16	−1.07 ± 0.26	−5.01 ± 4.9	−0.784 ± 0.09	−1.14 ± 0.36	−0.93 ± 0.37	−3.50 ± 1.51
**IC_50_, nM**	0.58 ± 0.19	2.29 ± 1.11	4.12 ± 1.31	4.77 ± 1.27	19.89 ± 1.16	6.58 ± 1.73	15.23 ± 1.34	5.65 ± 1.57	13.85 ± 1.20
**IC_50_ µg/L**	0.145 ± 0.05	0.579 ± 0.28	1.04 ± 0.33	1.20 ± 0.23	5.04 ± 0.29	1.43 ± 0.23	3.79 ± 0.33	1.40 ± 0.39	3.45 ± 0.29
**LOD** **µg/L**	0.012 ± 0.007	0.135	0.038 ± 0.022	0.037	3.24	0.08 ± 0.02	0.397	0.009	1.08
**LOQ** **µg/L**	-	-	0.24 ± 0.23	0.207	3.97	-	-	0.16	2.05
**R^2^**	0.999	0.997	0.98	0.85	0.77	0.999	0.983	0.77	0.89
	**Chloramphenicol**					**Estradiol**			
**Signalmax**	102.6 ± 8.8	98.8 ± 2.8	-	98.2 ± 3.8	97.2 ± 6.5	99.8 ± 0.5	97.7 ± 1.7	89.9 ± 6.7	96.1 ± 4.3
**Signalmin**	8.8 ± 1.2	28.7 ± 3.9	-	1.5 ± 3.4	20.0 ± 10.5	0.6 ± 0.7	−0.05 ± 2.39	0.45 ± 8.2	20.4 ± 6.5
**Slope**	−0.60 ± 0.05	−1.18 ± 0.32	-	−1.30 ± 0.19	−2.86 ± 1.99	−1.01 ± 0.07	−1.23 ± 0.15	−1.32 ± 0.62	−9.56 ± 4.3
**IC_50_, nM**	0.59 ± 0.05	10.25 ± 1.28	-	3.47 ± 1.15	12.91 ± 1.36	4.01 ± 0.74	9.67 ± 1.11	35.4 ± 1.36	21.83 ± 1.05
**IC_50_ µg/L**	0.192 ± 0.09	3.00 ± 0.41	-	1.12 ± 0.37	4.17 ± 0.44	1.09 ± 0.20	2.63 ± 0.30	9.72 ± 0.37	5.94 ± 0.28
**LOD** **µg/L**	0.004 ± 0.01	0.453	-	0.18	1.89	0.11 ± 0.03	0.362	0.41	4.60
**LOQ** **µg/L**	-	-	-	0.36	2.70	-	-	1.93	5.18
**R^2^**	0.997	0.988	-	0.94	0.72	0.999	0.998	0.90	0.82

*^1^ PBST; *^2^ sea water.

## Supplementary Materials

The following are available online at https://www.mdpi.com/1424-8220/20/19/5532/s1, Figure S1: 2D checkerboard titration experiments for 4e-BSA/As87, SA2-BSA/As155, CA6-BSA/As226 and 6E2_4-BSA/R64 combinations obtained in flow mode.
