# Supplementary material for: Multiplexed Immunosensor Based on the Amperometric Transduction for Monitoring of Marine Pollutants in Sea Water
Source: Sensors (Basel). 2020 Sep 27;20(19):5532. doi: 10.3390/s20195532 (PMC7584025; doi:10.3390/s20195532)

Multiplexed immunosensor based on the amperometric transduction for monitoring of marine pollutants in sea water

(SUPPORTING INFORMATION)

J.-Pablo Salvador 1,2, Klaudia Kopper1,2, Andrea Miti1,2, Ana Sanchis1,2 and M.-Pilar Marco1,2*

1 CIBER de Bioingeniería, Biomateriales y Nanomedicina (CIBER-BBN)

2 Nanobiotechnology for Diagnostics group (Nb4D). IQAC- CSIC. Jordi Girona 18–26, 08034 Barcelona, Spain.

** Correspondence: e-mail: jpablo.salvador@cid.csic.es*

Figure S1. 2D checkerboard titration experiments for 4e-BSA/As87, SA2-BSA/As155, CA6-BSA/As226 and 6E2-BSA/R64 combinations obtained in *flow mode (see Section 2.6)*.

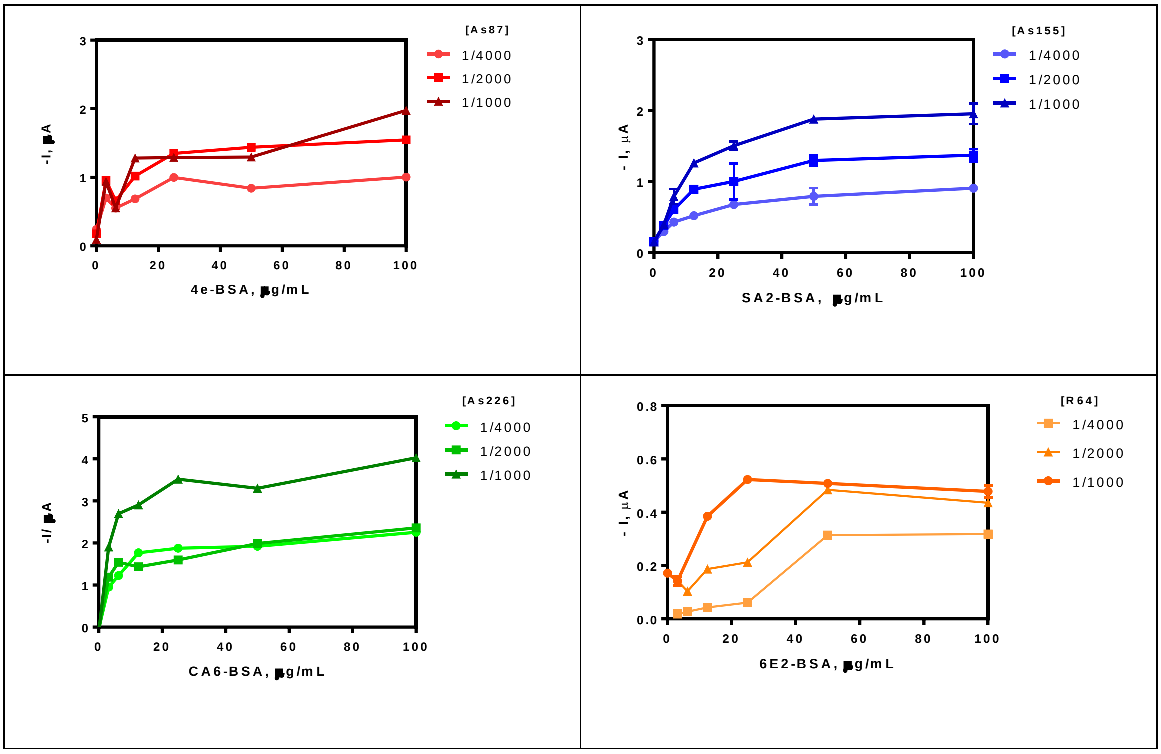

Supplement: Supplementary file 1 [file sensors-20-05532-s001.pdf]
